# Damage-associated molecular patterns (DAMPs) related to immunogenic cell death are differentially triggered by clinically relevant chemotherapeutics in lung adenocarcinoma cells

**DOI:** 10.1186/s12885-020-06964-5

**Published:** 2020-05-26

**Authors:** José Ignácio Gonzalez Solari, Eduardo Filippi-Chiela, Emily Salles Pilar, Vitória Nunes, Esteban Alberto Gonzalez, Fabrício Figueiró, Cristiano Feijó Andrade, Fábio Klamt

**Affiliations:** 1 Programa de Pós-Graduação em Ciências Pneumológicas, Laboratório de Pulmão e Vias Aéreas, FAMED/UFRGS, Hospital de Clínicas de Porto Alegre, Porto Alegre, Brazil; 2 Departamento de Ciências Morfológicas, Instituto de Ciências Básicas da Saúde, Universidade Federal do Rio Grande do Sul, Porto Alegre, RS Brazil; 3 Hospital de Clínicas Porto Alegre, Unidade de Pesquisas Experimental (Laboratório de Células, Tecidos e Genes), Porto Alegre, Brazil; 4 Programa de Pós-Graduação em Ciências Biológicas: Bioquímica, Universidade Federal do Rio Grande do Sul, Porto Alegre, Brazil; 5 Departamento de Bioquímica, Instituto de Ciências Básicas da Saúde, Universidade Federal do Rio Grande do Sul, Porto Alegre, RS Brazil; 6 Laboratório de Bioquímica Celular, Departamento de Bioquímica, Instituto de Ciências Básicas da Saúde, Universidade Federal do Rio Grande do Sul, Porto Alegre, RS Brazil

**Keywords:** Non-small cell lung carcinoma (NSCLC), Damage-associated molecular patterns (DAMPs), Immunogenic cell death (ICD), Autophagy, Cisplatin, Etoposide

## Abstract

**Background:**

Chemotherapeutics can stimulate immune antitumor response by inducing immunogenic cell death (ICD), which is activated by Damage-Associated Molecular Patterns (DAMPs) like the exposure of calreticulin (CRT) on the cell surface, the release of ATP and the secretion of High Mobility Group Box 1 (HMGB1).

**Methods:**

Here, we investigated the levels of ICD-associated DAMPs induced by chemotherapeutics commonly used in the clinical practice of non-small cell lung cancer (NSCLC) and the association of these DAMPs with apoptosis and autophagy. A549 human lung adenocarcinoma cells were treated with clinically relevant doses of cisplatin, carboplatin, etoposide, paclitaxel and gemcitabine. We assessed ICD-associated DAMPs, cell viability, apoptosis and autophagy in an integrated way.

**Results:**

Cisplatin and its combination with etoposide induced the highest levels of apoptosis, while etoposide was the less pro-apoptotic treatment. Cisplatin also induced the highest levels of ICD-associated DAMPs, which was not incremented by co-treatments. Etoposide induced the lower levels of ICD and the highest levels of autophagy, suggesting that the cytoprotective role of autophagy is dominant in relation to its pro-ICD role. High levels of CRT were associated with better prognosis in TCGA databank. In an integrative analysis we found a strong positive correlation between DAMPs and apoptosis, and a negative correlation between cell number and ICD-associated DAMPs as well as between autophagy and apoptosis markers. We also purpose a mathematical integration of ICD-associated DAMPs in an index (IndImunnog) that may represent with greater biological relevance this process. Cisplatin-treated cells showed the highest IndImmunog, while etoposide was the less immunogenic and the more pro-autophagic treatment.

**Conclusions:**

Cisplatin alone induced the highest levels of ICD-associated DAMPs, so that its combination with immunotherapy may be a promising therapeutic strategy in NSCLC.

## Background

Lung cancer is the leading cause of cancer mortality worldwide [1]. These malignant tumors have two main clinical types: small cell lung cancer (SCLC) and non-small cell lung cancer (NSCLC) [2]. NSCLC is the most common type, accounting for approximately 85% of all lung cancers. Adenocarcinoma, squamous cell carcinoma and large cell carcinoma constitute the subtypes of NSCLC [3]. This disease has a poor prognosis due to late diagnosis, resistance to chemotherapy and complications in advanced stages including high rates of metastasis, with an overall 5-year survival rate from 10 to 15% [1, 4].

Current traditional chemotherapy for lung cancer is not curative and provides limited benefits, with an average survival of less than 1 year [5]. Adding some optimism to this scenario, we have faced a decade of significant progresses in the identification of new biomarkers and immunotherapy [6]. Advances in cancer management and in key aspects of tumor biology have raised the concept that chemotherapy can induce tumor regression through different cell death mechanisms including non-immunogenic cell death, Immunogenic Cell Death (ICD), necrosis, among others [7]. Among the cell death mechanisms that have been described as immunogenic are apoptosis, necroptosis and pyroptosis [8, 9]. In addition, autophagy has also been described as contributing to anti-cancer immune response, but can also contribute to the resistance of cancer cells to therapy [10]. Understanding the crosstalk among these mechanisms and how cells resist cell death is fundamental to improve therapy efficacy [11]. Recent studies have demonstrated that some chemotherapeutics like doxorubicin, oxaliplatin, bleomycin and mitoxantrone as well as other non-chemical therapies like irradiation, photodynamic therapy and high hydrostatic pressure induce ICD in cancer cells [12–16].

ICD is characterized by the release of molecular signals, which are in general referred to as Damage-Associated Molecular Patterns (DAMPs) [9, 17]. The most relevant ICD-associated DAMPs considering the clinical response of cancer cells are (1) the pre-apoptotic exposure of chaperones on the cell surface - mainly calreticulin (CRT); (2) the secretion of adenosine triphosphate (ATP); and (3) the post-apoptotic release of High Mobility Group Box 1 protein (HMGB1) to the environment [18–20]. The abovementioned DAMPs (CRT, ATP and HMGB1) bind to specific receptors in the surface of dendritic cells: CD91, Toll-Like Receptors 4 and 2, and RAGE (Receptor for Advanced Glycation End products), respectively. The last one, is the most important receptor of HMGB1, being highly expressed in normal tissues, especially in lung [21]. In lung cancer microenvironment, binding of HMGB1 to RAGE activates immune cells [22]; on the other hand, it can also contribute to cancer cell proliferation and invasion [23, 24]. Considering their role in immune response, binding of these DAMPs to their receptors can also promote: (a) the engulfment of dying cells, (b) the presentation of tumor antigens and (c) the production of interleukins like IL-1β [19, 25–27]. All these signals are responsible for triggering the elicitation of the immune response targeted to tumor cells and the establishment of an antitumor immunological memory [28, 29].

The recruitment of immune cells to the tumor microenvironment and their reactivation to attack the cancer is a promising approach in lung cancer therapy [16]. Chemotherapeutics can induce ICD, thus contributing to the first step of this strategy (i.e. the enrichment of immune cells in tumor microenvironment). Therefore, it is fundamental to determine the capacity of classic chemotherapeutics used in the clinical management of NSCLC to induce the release of ICD-associated DAMPS by NSCLC cells. This may allow the rational design of clinical schedules combining these drugs with immunomodulators.

Here, we evaluated the induction of ICD-associated DAMPs by cisplatin, etoposide, carboplatin, paclitaxel and gemcitabine, both isolated and using clinically relevant co-treatments, in human lung adenocarcinoma cells through an integrative way including the analysis of apoptosis and autophagy. Since ICD-associated DAMPs indicate different steps of the cell death process, we also raised the concept that the integrative analysis of these DAMPs is quite important in the study of ICD. We also check for the influence of CRT and HMGB1 levels in the survival of patients from the TCGA databank.

## Methods

### Cell line and cell maintenance conditions

All experiments were performed using A549 human lung adenocarcinoma cell line, obtained from NCI-Frederick Cancer DCTD cell line repository in 2011. This cell line does not require ethics approval for their use in basic research. Personal cell stock in the − 80 freezer has been maintained and renewed. Experiments were performed in exponential growing cells never exceeding P20. Cells tested negatively for Mycoplasma in the beginning and the end of the experimental procedures. These cells were maintained in humidified incubator with RPMI 1640 medium supplemented with 10% fetal bovine serum (FBS) (Gibco/Invitrogen, São Paulo, SP Brazil), added with 2 mM of glutamine, 1% of penicillin/streptomycin, and 0.1% of amphotericin B (Sigma-Aldrich, St Louise, MO, USA) at 37 °C and 5% CO_2_.

### Chemotherapeutics treatments

A549 cells were seeded in 12-well plates (1 × 10^4^ cells/well). After 24 h of incubation, cells were treated for 48 h with cisplatin (Cis), etoposide (Eto), carboplatin (Carb), paclitaxel (Pac), gemcitabine (Gem) and also with co-treatments of Cis plus Eto and Carb plus Pac. Drugs concentration was chosen through dose-response curves based on the literature and on the peak of blood concentration reached by each drug. DMSO was used to dilute all chemotherapeutics except Carb, which was diluted in PBS. All chemotherapeutics were obtained from Sigma-Aldrich (St. Louis, MO, USA).

### Analysis of apoptosis by Annexin V-FITC and PI staining

Apoptosis was measured 48 h after treatment with Annexin V-FITC plus Propidium Iodide (PI) co-staining according to the manufacturer’s protocol (BD Biosciences; CA, USA). Briefly, cells were collected and washed once with Annexin binding buffer. After this, cells were resuspended in a staining buffer containing 2.5 μL of Annexin V-FITC and 5 μL of PI per sample, for 15 min in the dark. Cells were analyzed by flow cytometry (Attune-AB Applied Biosystems).

### Calreticulin (CRT) externalization measurement – flow cytometry

After the treatment, cells were collected and washed twice with ice cold PBS and fixed with 4% paraformaldehyde in ice cold PBS for 5 min. Cells were centrifuged at 1200 rpm for 5 min and washed again in ice cold PBS. Then cells were incubated for 30 min at 4 °C with staining solution (mix *per* sample: 200 μL ice cold PBS + 4 μL Fetal Bovine Serum + 1 μL Anti-CRT antibody #FMC75 [Abcam Cambridge, MA]). Isotype-control IgG1 (BD Biosciences; CA, USA) was used as control (mix *per* sample: 200 μL ice cold PBS + 4 μL fetal bovine serum + 1 μL isotype-control IgG1). Next, cells were washed twice with ice cold PBS, centrifuged for 5 min at 1200 rpm and resuspended in ice cold PBS. Samples were analyzed by flow cytometry (Attune-AB Applied Biosystems) to identify the percentage of CRT positive cells (i.e. cells that externalized the CRT) and also the intensity of CRT. We included a positive control based on the treatment of HCT116 colorectal cancer cells treated with Oxaliplatin [30].

### ATP release assay

To measure levels of extracellular ATP released in response to chemotherapeutics, supernatants were collected 48 h after treatment. We used the ATP assay kit Sigma-Aldrich (St. Louis, MO, USA) based on luciferin-luciferase conversion, according to the manufacturer’s protocol. Briefly, the supernatant was centrifuged at 1200 rpm for 5 min and 10 μL of cleared supernatants of each condition were transferred to a 96 well plate. Then, 90 μL of ATP reagent was added to each well, followed by incubation for 1 min at room temperature. After this, we analyzed fluorescence emission in a spectromax M3 microplate reader (Molecular Devices, Sunnyvale, CA, USA). We also included a positive control using HCT116 colorectal cancer cells treated with oxaliplatin [30].

### Extracellular HMGB1 measurement

To measure extracellular HMGB1, supernatants were collected and centrifuged at 1200 rpm for 5 min and immediately analyzed by *dot blot* immunoassay. Serial dilutions of samples (1, 2, 4, and 8 μL) were applied to a nitrocellulose membrane. The membrane was blocked with 5% BSA in TBS-T buffer (0.05% Tween20 in TBS) for 1 h at room temperature and incubated with primary antibody anti-HMGB1 (Abcam Cambridge, MA) 1:1000 dissolved in BSA/TBS-T for 30 min. Then, membrane was washed three times with TBS-T by 5 min and incubated with secondary antibody anti-rabbit 1:1000 (Abcam Cambridge, MA) for 30 min at room temperature. Next, membrane was washed three times with TBS-T (15, 5 and 2 min) and once with TBS (20 mMTris-HCl 150 mMNaCl pH 7.5) by 5 min, followed by incubation with Immobilon™ to chemiluminescent reaction (Millipore, EUA) for 1 min. Images of the membrane were recorded by the Image Quant imager LAS 500 (Healthcare GE Life Sciences). Quantification was performed using ImageJ software and levels of HMGB1 were corrected by cell number.

### Acridine Orange (AO) assay

Late step of autophagy was determined by detecting autolysosome formation through the Acridine Orange (AO) staining [31]. AO is a marker of acidic vacuolar organelles that fluoresces green in the whole cell (indicated by BL1 channel in flow cytometry) and red in acidic compartments (indicated by BL3 channel in flow cytometry), mainly in autolysosomes. Treated cells were trypsinized, collected and stained for 15 min at room temperature, in the dark, with AO at 2.7 μM. The percentage of AO-positive cells and the intensity of red/green fluorescence were assessed by flow cytometry (Attune-AB applied biosystems).

### Active Caspase 3 assay

To measure active caspase 3 in A549 cells we used the PE Active Caspase-3 Apoptosis Kit (BD Pharmingen), according to manufacturer’s instructions. Briefly, A549 cells were seeded in 24-well plates and grown to semi-confluence. Then, cells were treated with chemotherapeutics for 48 h, after which the culture medium and cells were centrifuged at 400 x g for 6 min. Then cells were washed twice with PBS 1x and resuspended in BD Cytofix/Cytoperm™ solution at a concentration of 3 × 10^5^ cells per 100 μl and incubated for 20 min at 4 °C. Afterward, the cells were washed twice with BD Perm/Wash™ buffer (1 ×) at room temperature. Finally, the cells were incubated in BD Perm/Wash™ buffer (1 ×) plus an antibody against active caspase-3 for 30 min at room temperature in the dark. Stained cells were analyzed by flow cytometry (Attune cytometry, BD biosciences). Cisplatin 80 μM was used as a positive control [32, 33].

### Immunocytochemistry to MAP 1LC3 and Calreticulin (CRT)

The cells were fixed in PA methyl alcohol for 5 min. Protein blocking with 3% BSA was performed for 1 h at room temperature. Cells were permeabilized with 0.05% Tween 20 diluted in PBS. Primary antibody incubation was overnight at 4 °C, using the following dilutions: Calcreticulin PE-conjugated (abcam) 1:50 and LC3B (abcam) 1: 400. Incubation with secondary antibody to LC3B was performed for 1 h 30 min at room temperature with goat anti-rabbit IgG secondary antibody, F (ab ‘) 2-FITC (Santa Cruz Biotechnology), at 1: 200. The slides were mounted with Fluoroshield mounting medium with DAPI (Abcam).

After image acquisition, we quantified levels of autophagy through counting the number of cells with at least 5 green dots in the cytosol using ImageJ [34].

CRT intensity was measured using Image Pro Plus 6.0 software (Media Cybernetics). We also assessed the distribution of CRT staining using the ‘margination’ tool of Image Pro Plus 6.0, which measure ‘the relative distribution of object intensity between center and margin. A value of 0.33 indicates a homogeneous object’. Margination value decreases as the distribution of the fluorescence increases in the periphery of the object.

### Nuclear morphometric analysis (NMA)

Nuclear morphometric analysis was performed as described by our group [35] to screen cell fate (i.e. apoptosis, senescence or mitotic catastrophe) based on nuclear shape and size. Briefly, treated cells were fixed with 4% paraformaldehyde and stained with DAPI 300 nM at room temperature in the dark. Images were acquired in a fluorescence microscope, followed by analysis in the Image Pro Plus 6.0 software (IPP6, Media Cybernetics). The nuclear contours were delimited using the magic wand tool, followed by the acquisition of following variables: Area, Radiusratio (Rr), Roundness (Rou), Aspect (Asp) and Areabox (Arbx). After the acquisition, data ware pasted in a spreadsheet available at www.ufrgs.br/labsinal/NMA, in which an analysis of nuclear area versus shape is performed. Nuclear shape is defined by the Nuclear Irregularity Index (NII), which is calculated by the following formula: NII = Asp−Arbx+Rr + Rou. Through this analysis, nuclei are classified according to the size and shape in the following populations: normal (N), small and regular (SR), small and irregular (SI), large and regular (LR), large and irregular (LIr). SR nuclei typically correspond to apoptotic cells, while LR and LIr correspond to nuclei from senescent cells.

### Statistical analysis

All statistics were performed in PASW Statistics 18. Shapiro-Wilk was used to test the normal distribution of results. Data are expressed as means ± standard error of the mean (SEM). Comparisons between groups were performed using the Student’s t-test and the One-Way Analysis of Variance (ANOVA) followed by Tukey *post-hoc* test for multiple comparisons, as appropriated. A ‘*p* value’ under 0.05 was considered as significant. Principal Component Analysis was used to determine the mathematical formula of the Index of Immunogenicity.

## Results

### Chemotherapeutics induce different rates of apoptosis in A549 cells

**Fig. 1:**
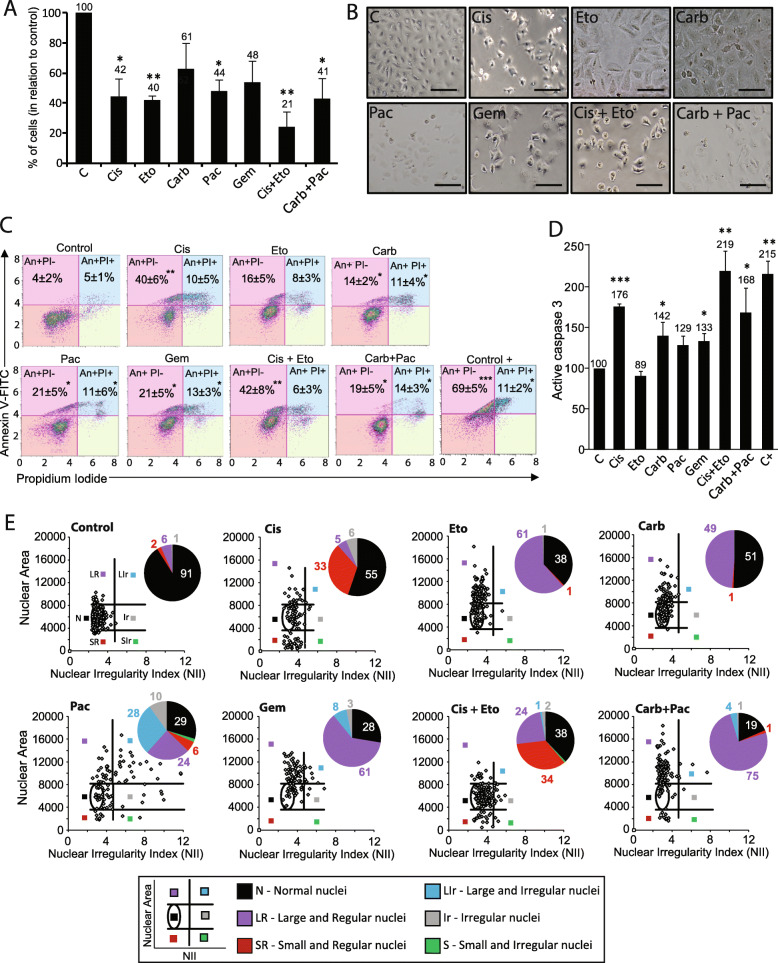
Induction of apoptosis and phenotypic alterations in NSCLC cells by classic chemotherapeutics. **a** Effect of chemotherapeutics in the number of A549 cells after 48 h of treatment. C – control (DMSO not exceeding 0.1%); Cis – Cisplatin 40 μM; Eto – Etoposide 13.2 μM; Carb – Carboplatin 200 μM; Pac – Paclitaxel 100 nM; Gem – Gemcitabine 0.96 μM; Cis + Eto – Cisplatina 40 μM + Etoposide 13.2 μM; Carb+Pac – Carboplatin 200 μM + Paclitaxel 100 nM. Cell number was analyzed by cell counting in a flow cytometer. Data represent the mean ± SEM;*p < 0.05 and **p < 0.01 in relation to the control considered as 100%, using ANOVA (Tukey *post-hoc*). **b** Representative images of cells treated with chemotherapeutics for 48 h. Images were obtained by phase-contrast inverted microscope (200x). **c** Effect of chemotherapeutics on the induction of apoptosis and necrosis after 48 h in A549 cells. Dot blot graphs show annexin V-FITC/ PI co-staining with quadrant analysis, as following: early apoptotic cells (upper left), late apoptotic cells (upper right), plasma membrane permeabilization (low right). Results are expressed as the percentage of cells in each quadrant ± SEM; *p < 0.05 and **p < 0.01 in relation to the control, using ANOVA (Tukey *post-hoc*). C+: A549 cells treated with Cis 80 μM. **d** Levels of active caspase 3 measured through flow cytometry. Data represent the mean ± SEM;*p < 0.05 and **p < 0.01 in relation to the control considered as 100%, using ANOVA (Tukey *post-hoc*). Dot plots are shown in Fig. S1D. C+: A549 cells treated with Cis 80 μM. **e** Nuclear Morphometric Analysis. Dot blots represent Nuclear area versus Nuclear Irregularity Index (NII). Dots in each condition correspond to single nuclei (at least 50 nuclei are shown to each condition). Nuclear phenotype populations are shown in the legend on the bottom, and the percentage of nuclei in each population is shown in the pie chart. Representative figures of DAPI-stained nuclei are shown in Fig. S1C

We initially did a dose-response curve to each drug to determine the doses of chemotherapeutics necessary to reach toxicity near to IC_50_ after 48 h of treatment in A549 cells (Additional file 1: Fig. S1). We then choose the following doses to the next steps of the study: Cis 40 μM; Eto 13.2 μM; Carb 200 μM; Pac 100 nM; Gem 0.96 μM. The effect of these treatments and co-treatments of Cis + Eto and Pac + Carb in cell counting after 48 h is shown in Fig. 1a. Cis *(p = 0.023)*, Eto *(p = 0.008)*, Pac *(p = 0.021)*, Cis + Eto *(p = 0.002)* and Carb+Pac *(p = 0.025)* reduced cell number. We then search for the mechanisms involved in this response.

Apoptosis is the most known mechanism of programmed cell death [36]. Several chemotherapeutics trigger apoptosis through the intrinsic pathway, which involves the release of pro-apoptotic mitochondrial factors, activation of caspases and cleavage of cellular components by these proteases [37]. As a consequence, morphological markers of early apoptosis include cell shrinkage, nuclear condensation and membrane blebbing [38, 39]. Late apoptosis, in turn, is characterized by nuclear and cellular fragmentation [40]. An increase in the number of cells with alterations that resemble apoptosis was observed, including cell detachment and shrinkage, in higher extent to Cis, Gem and Cis + Eto, and lower to Eto (Fig. 1b). In agreement to this, we observed a drug-specific increase in the proportion of cells with reduced area and increased intracellular granularity (Additional file 2: Fig. S2A **–** note the differential increase of the population 3, suggestive of early apoptosis, depending on the treatment). On the other hand, we also observed an increase in large and complex cells (i.e. high FSC and SSC) in response to other treatments, such as Eto. By microscopy, we confirmed that some treatments reduced cell area (Cis + Eto *p = 0.041*), while Eto (*p = 0.016*) and Gem *(p = 0.011)* increased it (Additional file 2: Fig. S2B).

Then, we determined the apoptosis rate in A549 cells in response to each treatment through specific assays. We observed that Pac *(p = 0.031)*, Gem *(p = 0.031)*, Carb+Pac *(p = 0.038)* and, in higher extent, Cis *(p = 0.008)* and Cis + Eto *(p = 0.009)* increased the percentage of early apoptotic cells (Annexin^+^/PI^−^). Pac *(p = 0.042),* Gem *(p = 0.031),* Carb *(p = 0.035)* and Carb+Pac *(p = 0.028)* also increased the percentage of late apoptotic cells (Annexin^+^/PI^+^) (Fig. 1c). When assessing active caspase 3, we found an increase in the number of small cells with active caspase 3 (Additional file 2: Fig. S2D) and an increase in the levels of active caspase 3 in relation to control to all treatments (Cis *p = 0.001*; Carb *p = 0.041*; Gem *p = 0.039*; Cis + Eto *p = 0.009*; Carb+Pac *p = 0.017*); C+ *p = 0.007*), except Eto (Fig. 1d). Among all treatments, Cis and Cis + Eto showed the higher levels of active caspase 3.

Finally, we did the nuclear morphometry analysis (NMA) to infer different cell fates (i.e. apoptosis, senescence and mitotic catastrophe) based on nuclear morphometry [35]. In NMA, shape and area are obtained for single nuclei, which are classified according to their morphometry in different populations as shown in the legend of Fig. 1e. Representative images of nuclei of A549-treated cells are in Additional file 2: Fig. S2C. We found an increase in ‘small and regular’ (SR) nuclei after Cis *(p < 0.0001)* and Cis + Eto *(p < 0.0001)* treatments, suggesting early apoptosis. Gem *(p < 0.0001)*, Eto *(p < 0.0001),* Carb *(p = 0.0016)* and Carb+Pac *(p < 0.0001)* increased the percentage of ‘large and regular’ (LR) nuclei, suggesting cell cycle arrest and/or cell swelling, confirming data from cell area. Pac *(p < 0.0001)* increased the nuclear irregularity, an effect that was attenuated in the Pac + Carbo co-treatment (Fig. 1e and Additional file 2: Fig. S2C). Altogether, these results suggest that Cis was the most pro-apoptotic drug, while Eto induced the lower levels of apoptosis in A549 cells. Gem and Eto increased cell and nuclear size which was not accompanied by high levels of apoptosis markers. Since we observed reduced cell counting in relation to control, our data suggest that these treatments could be triggering cell cycle arrest or a non-apototic cell death mechanism, such as pyroptosis. Finally, Pac induced nuclear alterations that resemble mitotic catastrophe, which was mitigated in the combination of Carb+Pac.

### Autophagy is high in response to chemotherapeutics that induce low apoptosis

**Fig. 2:**
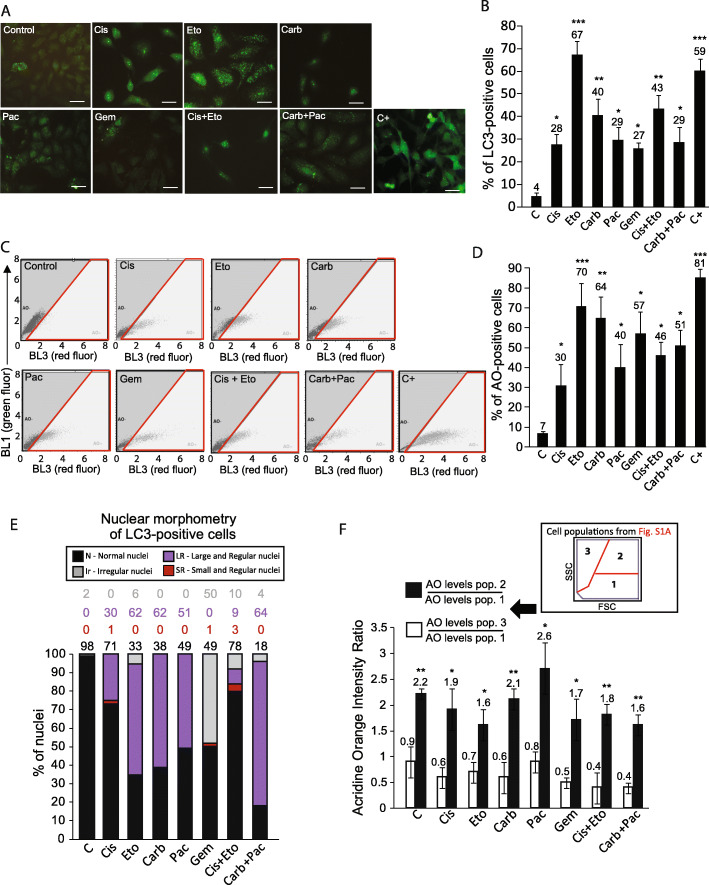
Induction and role of autophagy in the response of A549 cells to chemotherapeutics. Cells were treated with chemotherapeutics for 48 h with doses cited in the text, followed by analysis of autophagy markers. **a** Representative images of LC3 immunocytochemistry. **b** Percentage of LC3-positive cells. Cells with at least 5 green dots were considered to be positive. U87 glioma cells treated with Rapamycin 100 nM for 24 h were used as a positive control. **c** Representative plots of Acridine orange (AO) staining. Green (BL1) x red (BL3) dot plot of AO-stained cells. Cells inside the red area were considered as positive. **d** Percentage of AO-positive cells. U87 glioma cells treated with Rapamycin 100 nM for 24 h were used as a positive control. Data represent the percentage of AO-positive cells ± SEM;*p < 0.05; **p < 0.01 and ***p < 0.001, using ANOVA (Tukey *post-hoc*). **e** Nuclear morphometry of LC3-positive cells. Nuclear Morphometric Analysis (NMA) was performed in LC3-positive cells. The percentage of nuclei in each population of NMA is shown. **f** Acridine orange intensity ratio calculated to cell populations from Fig. S1A. We compared the intensity of AO staining in cells from the population 1 (cells with normal phenotype based on FSC x SSC) with the intensity of AO staining cells from the population 2 (viable cells with high intracellular complexity) and population 3 (early apoptosis morphology). *p < 0.05, **p < 0.001 - black bar in relation to the correspondent white bar

Autophagy is a mechanism through which cells digest their old, malfunctional or damaged components in order to maintain or restore the homeostasis. It involves the dynamic formation of organelles called autophagosomes engulfing the components to be degraded, which are delivered to lysosomes [41, 42]. Data from flow cytometry showed that some treatments increased intracellular granularity measured by SSC, suggesting autophagy induction (Additional file 2: fig. S2A). To confirm this, we immunostained treated cells with anti-LC3 antibody to measure early step of autophagy (i.e. the formation of autophagosomes). Figure 2a Shows representative images of LC3 immunostaining. After treatments, we found an increase in the percentage of LC3-positive cells (i.e. cells with at least 5 green dots in the cytosol) to all conditions, in higher extent to Eto (Cis *p = 0.031*; Eto *p < 0.001*; carb *p = 0.009*; Pac *p = 0.030*; gem *p = 0.012*; Cis + Eto *p < 0.008*; carb+Pac *p = 0.032*; C+ *p < 0.001*) (Fig. 2a and b). To confirm this, we stained cells with Acridine Orange (AO), a fluorescent dye used to measure the late step of autophagy (i.e. the formation of autolysosomes) [31]. Figure 2c Shows the representative plots of AO flow cytometry to all conditions. Carb and, in higher extent, Eto induced the higher levels of AO staining, (Cis *p = 0.048*; Eto *p < 0.001*; carb *p = 0.007*; Pac *p = 0.043*; gem *p = 0.031*; Cis + Eto *p = 0.02*; carb+Pac *p = 0.021*; C+ *p < 0.001*) (Fig. 2d). These results, in association with apoptosis data, suggest that autophagy is activated in NSCLC cells exposed to all treatments, and that the higher the levels of autophagy, the low the levels of apoptosis

Indeed, autophagy has being associated with cell survival and resistance to treatment. We then test the impact of autophagy in cell fate through two single cell analyses. Firstly, we assessed the nuclear morphometry of all LC3-positive cells. We found that quite all LC3-positive cells showed normal, enlarged or irregular nuclei, but not small and regular nuclei (which is an indicative of apoptosis) (Fig. 2e and Additional file 3: fig. S3). Corroborating this, we compared AO levels in the three populations of cells showed in Additional file 2: fig. S2A. Through this, we found that shrinked cells (i.e. cells with small size, suggestive of early apoptosis) showed lower levels of AO staining than normal cells (Fig. 2f – white bars**)**, while cells with high intracellular granularity (i.e. high SSC) showed higher levels of AO than healthy cells (Cis *p = 0.031*; Eto *p = 0.04*; carb *p = 0.009*; Pac *p = 0.03*; gem *p = 0.029*; Cis + Eto *p = 0.036*; carb+Pac *p = 0.01*) (Fig. 2f – black bars). Altogether, these results suggest that autophagy may protect NSCLC cells from cell death.

### Chemotherapeutics differentially increase the level of Calreticulin (CRT) and promote its translocation to the plasma membrane of A549 cells

**Fig. 3:**
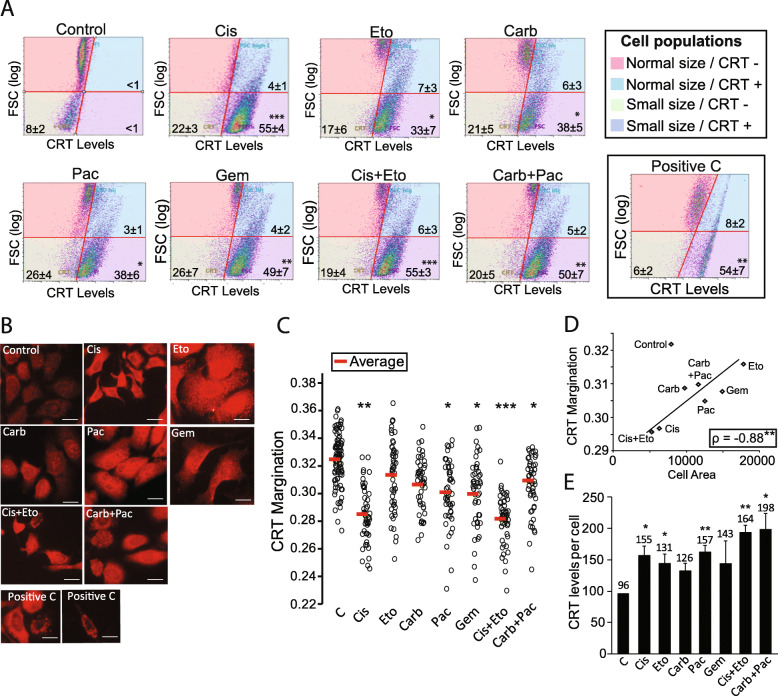
Levels of calreticulin (CRT) exposure and margination in A549 cells treated with chemotherapeutics. **a** Representative plots of FSC (cell size) versus levels of CRT exposure in the cell surface. Note that axes are in the log scale to allow the observation of shrinked cells (i.e. low FSC cells). Numbers represent the percentage of cells in each quadrant, as described in the legend on the right. On the right is also shown the positive control (CT116 colorectal cancer cells treated with oxaliplatin 25 μM for 48 h). **b** Representative images of CRT immunocytochemistry. **c** Margination of CRT. Each dot represents a single cell; red lines represent the average. **d** Correlation between cell area and CRT margination to the treatments tested. **e** Total level of CRT in A549 cells treated with chemotherapeutics, obtained from the intensity of red fluorescence measured using the Image Pro Plus 6.0 software. To all graphs from this figure, *p < 0.05; **p < 0.01 and ***p < 0.001 in relation to control (ANOVA followed by Tukey *post-hoc*)

The translocation of CRT from the ER to the plasma membrane is an early event in ICD. To measure this, we immunostained A549 treated cells to CRT, followed by flow cytometry. Important to mention, the protocol used here did not involve any permeabilization step, so that all CRT stained was exclusive from cell surface. After 48 h, all treatments triggered an increase in the translocation of CRT to the cell surface (Additional file 4: Fig. S4A). To relate CRT exposure to cell size, we next looked to the association between FSC (i.e. cell size) and level of CRT in the plasma membrane. We found that all treatments increased the percentage of small and CRT^+^ cells (Cis *p < 0.001*; Eto *p = 0.019*; Carb *p = 0.011*; Pac *p = 0.013*; Gem *p = 0.008*; Cis + Eto *p < 0.001*; Carb+Pac *p = 0.008*; C+ *p = 0.006*) (Fig. 3a – bottom right quadrant; Additional file 4: Fig. S4B). Cis induced the highest increase in this percentage, while Eto induced the lowest one. It is important to note that in control group, shrinked cells were negative to CRT^+^, suggesting that basal cell death does not involve the externalization of CRT, in opposite to chemotherapeutics-induced cell death. To confirm flow cytometry data, we also immunostained CRT in attached cells. Using the Image Pro Plus software, we measured CRT margination, i.e. the distribution of CRT from the center to the periphery of single cells. We also associated this variable with cell area in single cells. Representative images of CRT immunocytochemistry are shown in Fig. 3b. We observed an increase in CRT margination to Cis *(p = 0.009)*, Pac *(p = 0.024)*, Gem *(p = 0.025)*, Cis + Eto *(p < 0.001)* and Carb+Pac *(p = 0.043)* (Fig. 3c and d). Furthermore, we found a strong negative correlation between cell area and CRT margination considering all treatments, both to single cells (Additional file 4: Fig. S4C) and to the cell population in average *(p = 0.008)* (Fig. 3d). This data suggest that the smaller the cells, the higher the exposure of CRT in cell surface, which plays a role as an `eat me` signal to phagocytes. Interestingly, corroborating data from flow cytometry to CRT, this correlation was not observed in the control condition, suggesting that in basal conditions this `eat me` signal may not be so efficiently activated.

### Low levels of CRT are associated with poor prognosis and high tumor grade in NSCLC adenocarcinoma

Considering the potential of ICD-associated DAMPs as biomarkers of prognosis and response to therapy, we found that high levels of CRT are associated to a better prognosis in NSCLC (subtype: adenocarcinoma) in the TCGA cohort (Additional file 5: Fig. S5A). In grade i-iii tumors, levels of CRT were not associated with the fate of patients (Additional file 5: Fig. S5B and S5C to D). On the other hand, in grade iv tumors (i.e. metastatic) we observed an increase in the survival rate to patients showing tumors with high levels of CRT (Additional file 5: Fig. S5B and S5F). Altogether, these results suggest that high levels of CRT are associated to a better prognosis in lung cancer. In this sense, here we found that chemotherapeutics increased CRT levels in NSCLC cells (Cis *p = 0.033;* Eto *p = 0.04*; Pac *p = 0.01;* Cis + Eto *p = 0.008;* Carb+Pac *p = 0.017*) (Fig. 3e), which could contribute with a better prognosis to the patients.

### Chemotherapeutics increase ATP release and HMGB1 secretion in A549 cells

**Fig. 4:**
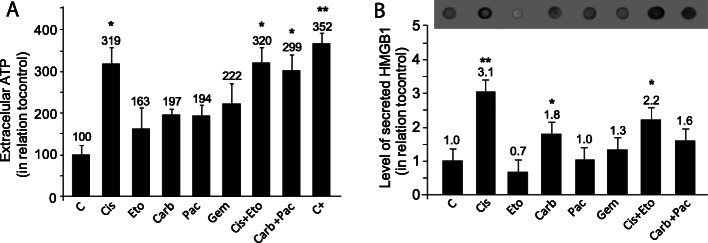
Levels of extracellular ATP and HMGB1 in the supernatant of A549 cells treated with chemotherapeutics. **a** Levels of extracellular ATP measured by colorimetric assay; data represent the mean ± SEM *p < 0.05, **p < 0.01, in relation to control considered as 100%, using ANOVA (Tukey *post-hoc*). HCT116 colorectal cancer cells treated with 25 μM of oxaliplatin for 48 h were used as positive control. **b** Levels of HMGB1 released by cell in culture medium. Data represent the mean ± SEM;*p < 0.05; **p < 0.01, in relation to control considered as 1, using ANOVA (Tukey *post-hoc*)

We next analyzed the levels of late-stage ICD-associated DAMPs, i.e. extracellular ATP and HMGB1, in A549-treated cells. Secreted ATP is well recognized as the ‘find me’ signal that attracts immune cells to tumor microenvironment. We found that among single treatments, only Cis increased ATP in the supernatant (*p = 0.012*), which was not increased by Eto adding (*p = 0.012*). The co-treament of Carb**+**Pac also increased the release of ATP (*p = 0.02*) (Fig. 4a). Carb (*p = 0.042*) and, in higher intensity, Cis (*p = 0.009*) and Cis + Eto (*p = 0.008*) increased the secretion of HMGB1 to the extracellular medium (Fig. 4b).

### Index of immunogenicity (IndImunnog) indicates that Cis triggered the highest levels of ICD-associated DAMPs in NSCLC cells

Next, we performed an integrative analysis using data from ICD-associated DAMPs, cell counting, apoptosis and autophagy measurements. All DAMPs correlated positively with each other and with apoptosis markers, while correlated negatively with cell number (Additional file 6: Fig. S6). We also observed that those treatments that decreased cell counting in higher extent showed also a robust increase in apoptotic cells and ICD-associated DAMPs, concomitant to lower levels of autophagy. Cis and its combination with Eto were the treatments that better represent this response profile. On the other hand, treatments that induced high levels of autophagy triggered the lowest levels of ICD-associated DAMPs and apoptosis markers. Eto is the condition that better represents this response.

**Fig. 5:**
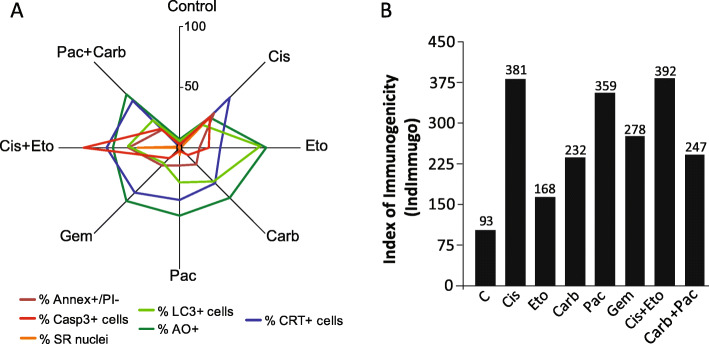
Integrative analysis of ICD-associated DAMPs, cytotoxicity and autophagy. **a** Radar plot integrating the cellular responses measured as percentage of cells in relation to total population. **b** Index of Immunogenicity calculated to each treatment. We used the weights provided by a Principal Component Analysis to propose the following mathematical formula to measure the immunogenic potential of each condition: 0.943*ATP + 0.791*HMGB1 + 0.9*CRT levels + 0.487*CRT Margination

Finally, having in mind that each DAMP has a specific role in the different stages of recruitment and immune activation, we propose here the generation of an index, here named Index of Immunogenicity (IndImmunog) to suggest the immunogenic potential of each treatment. To this, we performed a Principal Component Analysis (PCA) in SPSS to obtain the weights of each variable in the IndImmunog. To the PCA, we used the following variables: extracellular ATP, secreted HMGB1, CRT levels and CRT margination. After PCA, we obtained the following formula: IndImmunog = 0.943*ATP + 0.791*HMGB1 + 0.9*CRT levels + 0.487*CRT Margination. IndImmunog suggests that Cis was the most immunogenic treatment, while Eto was the least one (Fig. 5b).

## Discussion

The recruitment of immune cells to the tumor microenvironment is a therapeutic strategy that has shown promising results even in aggressive tumors like lung cancer. The capacity of chemotherapeutics to induce ICD is quite heterogeneous and depends on the cell type and the experimental conditions. Also, there are few studies that evaluated ICD-associated DAMPs from an integrated point of view and linked to other cell death and survival mechanisms. In the current study, we assessed the capacity of clinically relevant chemotherapeutics to induce ICD-associated DAMPs in NSCLC cells. All drugs tested here induced ICD at different levels. Cis was the most cytotoxic and triggered the highest levels of ICD-associated DAMPs, while Eto induced the lowest levels of these markers.

The prognostic role of ICD-associated DAMPs has been exploited in recent years. Confirming findings from others, we found that high levels of CRT were associated to a better prognosis in NSCLC in the TCGA cohort (data assessed using The Human Protein Atlas website) [43]. This association was stronger in high grade tumors, in which low levels of CRT were associated with higher risk of death. Mechanisms underlying this prognostic role may be related to the increased recruitment of immune cells to the microenvironment of tumors expressing high levels of CRT [44, 45]. CRT exposure in dying cells is the crucial ‘eat me’ signal during ICD, increasing the recognition of dying tumor cells by phagocytes, mainly dendritic cells [29]. CRT also induces an increase in the expression of adhesion molecules by endothelial cells, facilitating the infiltration of tumor-specific lymphocytes in the tumor microenvironment [46]. It is important to note that this data is based on calreticulin mRNA levels in a tumor biopsy, without considering the subcellular distribution of calreticulin in cancer cells. Thus, we cannot conclude that this higher survival is due to ICD. However, it is plausible to assume that tumor cells that have higher levels of expression of CRT have a greater capacity to translocate this chaperone to the cell surface in response to endoplasmic reticulum stress induced by chemotherapy. In this sense, here we found that basal cell death as observed in control was not associated with increase in CRT exposure, unlike chemotherapy-induced cell death. Indeed, the translocation of CRT from the ER to the cell surface is one of the main differences between immunogenic and non-immunogenic death and a key factor to ICD [20, 29]. Furthermore, here we found an increase of CRT levels in single cells after Cis, Eto, Pac, Cis + Eto and Pac + Carb treatment. This could lead to a better prognosis, as suggested by data from TCGA. In addition to this, the higher levels of CRT in cell surface and the increase of CRT margination induced by some treatments could increase the ‘eat me’ signal in tumor microenvironment, thus contributing to the elimination of therapy resistant cells.

After CRT exposure, late dying cells secret HMGB1, which has a dual role in lung cancer depending on whether it is intracellular or extracellular [22]. Extracellular HMGB1 has a paracrine role, promoting the processing and presentation of tumor antigens by dendritic cells [47, 48]. On the other hand, intracellular HMGB1 promotes growth, invasion and resistance of cancer cells to therapy, both in vitro and in vivo [49]. Assessing the TCGA database we found that high levels of HMGB1 are associated with poor prognosis in NSCLC, suggesting that the intracellular role is dominant (Additional file 7: Fig. S7). This dual role of HMGB1 also indicates that the evaluation of single ICD-associated DAMPs or the evaluation of total levels per se in a given sample not necessarily represents an accurate biological information, which could be improved through an integrated evaluation. Indeed, silencing of a unique protein, such as CRT or HMGB1, is enough to suppress ICD and immune activation in response to chemotherapy [27]. In this sense, here we purpose the use of an integrative index of DAMPs levels (IndImmunog) to suggest the immunogenic potential of a given treatment. Mathematical formula of IndImmunog was defined by Principal Component Analysis, and indicated that Cis and Pac may be the most immunogenic treatments. Eto, on the other hand, generated the lowest value of IndImmunog.

Considering the link between ICD and response to therapy, high expression of ICD-associated DAMPs correlated with the reduction of tumor size in response to classical chemotherapeutics [18, 50, 51]. ICD is crucial to the response of colorectal adenocarcinoma cells to oxaliplatin in mice [27]. Here, Eto induced low levels of ICD, as in colorectal cancer cells [29], while Cis induced the highest level of ICD, corroborating data from human melanoma cells and colorectal cells [52]. However, other studies have shown that Cis alone does not induce ICD in cancer cells [22, 29], possibly because the drug does not induce significant Endoplasmic Reticulum (ER) stress response, the main cellular mechanism triggering externalization of CRT [53]. The differential capacity of ICD induction by specific chemotherapeutics may also be due to differences in the genetic backgrounds of different tumor types, since the exposure of CRT is triggered by a complex pathway involving ER stress response, which is altered in some tumor types [14, 19].

Another relevant aspect is related to the different kinetics of ICD-associated DAMPs exposure in dying cells. We found that CRT exposure (pre-apoptotic ICD marker) increased during the shrinkage of dying cells, and then reduced in cells that reached their smallest size just before cell fragmentation. This may justify some data from Cis-treated colorectal cancer cells, which showed an increase in HMGB1 but no alteration in CRT exposure, probably because ICD-associated DAMPs were assessed in the context of late apoptosis so that the peak of CRT exposure was lost [27]. This kinetics may also explain the observation of increased CRT exposure and ATP secretion concomitant to no alteration in secreted HMGB1 (post-apoptotic ICD marker) after Cis for 24 h in NSCLC [26].

Understanding the cellular mechanisms involved in the response of cancer cells may allow the modulation of cell fate and, as a consequence, the response of immune cells in tumor microenvironment. Indeed, here we observed that a single treatment can trigger a multitude of cell responses in A549 cells. While Cis-treated cells were positive to all apoptosis markers, for instance, Eto-treated cells appeared enlarged, highly autophagic and negative to all apoptotic markers. Thus, it is plausible to assume that non-apoptotic cell fates were also triggered in some conditions. In this sense, Carb, Pac, Gem and Carb+Pac increased both PI incorporation and the area of attached cells. Cell swelling and membrane permeabilization are typical alterations of pyroptosis [54, 55], which can also be immunogenic [56, 57].

Another mechanism investigated here was autophagy, which acts as an anti-apoptotic mechanism in response to Eto, Cis and Gem in NSCLC [35, 40]. Autophagy is essential to the immunogenic release of ATP by dying cells in response to therapy [17, 37]. Corroborating this, the suppression of autophagosome formation (early autophagy) reduced CRT exposure in oxaliplatin-treated colorectal cancer cells [30]. On the other hand, the suppression of late steps of autophagy after ER stress [58–60] increased CRT exposure [30]. The dominant role of autophagy (i.e. whether avoiding cell death or contributing to the exposure of ICD-associated DAMPs) may depends on the intensity of damage, the autophagic capacity of the cell and the cellular components injured [10]. The current data suggest that the cytoprotective role of autophagy is dominant, allowing cells to eliminate or repair damaged cellular components. This hypothesis is reinforced by our data showing that autophagy was higher in those treatments that did not induce high levels of ICD-associated DAMPs, like Eto (Fig. 5a).

## Conclusions

Here we found that classical chemotherapeutics used in the clinical management of NSCLC vary on their capacity to induce ICD. ‘Immunogenic therapies’ induce cancer cells to release a set of molecules that recruit immune cells to the tumor microenvironment, increasing the possibility of activation of specific immune responses against cancer cells. In this scenario, potent ICD inducers like Cis could be rationally combined to strategies that reactivate immune cells to eliminate chemotherapy-resistant NSCLC cancer cells.

## Supplementary information

**Additional file 1: Fig. S1** Dose-response curves in A549 cells. Cells were treated with the chemotherapeutics as indicated, for 48 h. After this, cell number was determined through flow cytometry.

**Additional file 2: Fig.** S2 Morphological changes and caspase activation in A549 cells treated with chemotherapeutics. **(A)** Analysis of cell phenotype assessed by flow cytometry. Population 1 indicates normal cell morphology. Reduced cell size (low FSC) with increased intracellular granularity (high SSC) is suggestive of early apoptosis (population 3); high intracellular granularity (high SSC) suggests cytoplasmic vacuolization, as observed after autophagy activation (population 2); and cell debrie suggests late apoptosis, which is characterized by cell fragmentation (population 4). A scheme showing these populations is shown on the right. **(B)** Cell area measured through images from immunocytochemistry. **(C)** Representative images of nuclear staining with DAPI (magnification: 200x). **(D)** Representative plots of active caspase 3 versus FSC. Red area delimits the population of cells with small size (i.e. cell shrinkage, a typical change of early apoptotic cells) and active caspase 3. Numbers represent the percentage of cells inside the red area ± SEM. Cisplatin 80 μM was used as a positive control (C+).

**Additional file 3: Fig. S3** Nuclear and LC3 co-staining. Representative images from each treatment are shown. Double arrowheads: nuclei classified as small and regular in the NMA; Arrows – nuclei classified as large in the NMA; Single arrowhead: nuclei classified as normal in NMA.

**Additional file 4: Fig.** S4 Calreticulin (CRT) exposure and its correlation with cell area. **(A)** Representative histograms of cell count and CRT exposure in the cell surface, as obtained by flow cytometry. *p < 0.05, **p < 0.01 and ***p < 0.001 in relation to control. **(B)** Dot plots for CRT levels and cell size (FSC). **(C)** Correlation between CRT margination and cell area, measured from calreticulin immunocytochemistry (Fig. 3B). *p < 0.05, **p < 0.01 in relation to control.

**Additional file 5: Fig. S5** Influence of CRT levels in the prognosis and staging of NSCLC. (A) Kaplan-Meier survival curve to patients expressing high or low levels of CRT in the TCGA cohort. (B) Dead/live ratio of patients for grade i-iv tumors according to CRT levels. (C to F) Influence of CRT levels on the outcome (i.e. dead or alive) of patients with stage i to iv NSCLC.

**Additional file 6: Fig. S6** Correlation matrix to all measurements assessed. ρ coef. Represent the pearson correlation coefficients. Red and green boxes correspond to all negative or positive significant correlations, respectively. Abbreviatures: ‘% Annex+/PI- cells’ - % of annexin+/Propidium Iodide negative cells; ‘% CRT+ cells’ - % of calreticulin-positive cells; ‘% AO+ cells’ - % of acridine orange-positive cells; ‘ATP’ - levels of extracellular ATP; ‘HMGB1’ - levels of extracellular HMGB1; ‘CRT Marg.’ - Margination of Calreticulin; ‘%LR, %N, %SR and %Irreg’ - percentage of ‘Large and Regular’, ‘Normal’, ‘Small and Regular’ and ‘Irregular’ nuclei from NMA analysis; ‘Nuc Area’ - nuclear area.

**Additional file 7: Fig. S7** Influence of HMGB1 levels in the prognosis of NSCLC. Kaplan–Meier survival analysis according to HMGB1 levels.

## Data Availability

Unfortunately we cannot make raw data available at this moment due to two reasons: firstly, we are performing several additional analyses in raw data from flow cytometry and microscopy in order to develop some new protocols to objectively analyze data from these two techniques. In addition, this analysis involves intellectual protection and the registration of a patent. Once these side projects have been completed, the data will be promptly made available.

## Abbreviations

AO: Acridine Orange
ATP: Adenosine trihosphate
Carb: Carboplatin
CD91: Cluster of differentiation 91
Cis: Cisplatin
CRT: Calreticulin
DAMPs: Damage-Associate Molecular Patterns
*ER*: *Reticulum Endoplasmic*
Eto: Etoposide
Gem: Gemcitabine
HMGB1: High Mobility Group Box 1 protein
ICD: Immunogenic cell death
IL-1b: *Interleukin 1 beta*
NSCLC: Non-small cell lung cancer
Pac: Paclitaxel
PI: Propidium Iodide
SCLC: Small cell lung cancer
TLR2: Toll-like Receptor 2
TLR4: Toll-Like Receptor 4
