# Supplementary material for: Damage-associated molecular patterns (DAMPs) related to immunogenic cell death are differentially triggered by clinically relevant chemotherapeutics in lung adenocarcinoma cells
Source: BMC Cancer. 2020 May 26;20:474. doi: 10.1186/s12885-020-06964-5 (PMC7251700; doi:10.1186/s12885-020-06964-5)
Supplement: Supplementary file 1 — Additional file 1: Fig. S1 Dose-response curves in A549 cells. Cells were treated with the chemotherapeutics as indicated, for 48 h. After this, cell number was determined through flow cytometry. [file 12885_2020_6964_MOESM1_ESM.pdf]

Figure S1

Solari & Filippi-Chiela et al

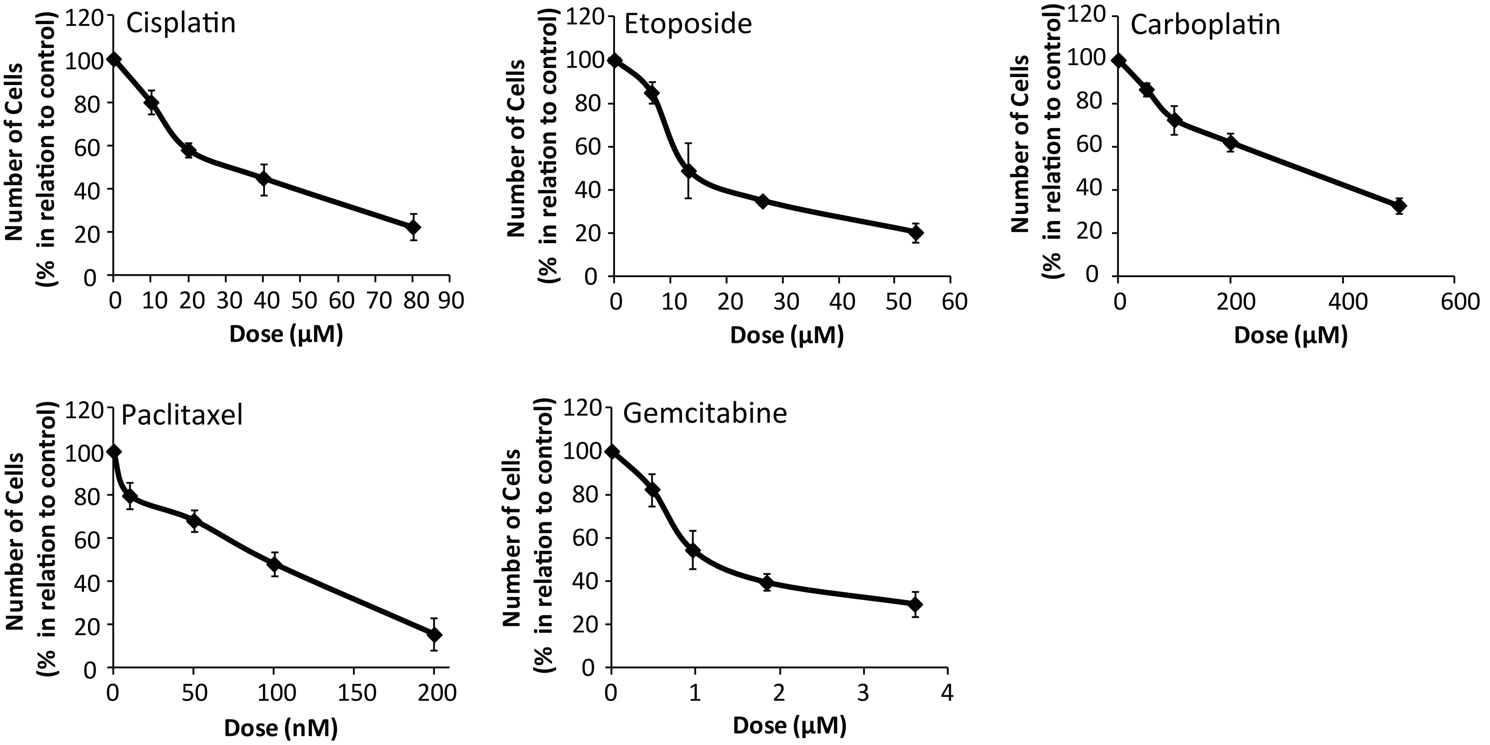

Figure S1. Dose-response curves in A549 cells. Cells were treated with the chemotherapeutics as indicated, for 48h. After this, cell number was determined through flow cytometry.
