# Supplementary material for: Damage-associated molecular patterns (DAMPs) related to immunogenic cell death are differentially triggered by clinically relevant chemotherapeutics in lung adenocarcinoma cells
Source: BMC Cancer. 2020 May 26;20:474. doi: 10.1186/s12885-020-06964-5 (PMC7251700; doi:10.1186/s12885-020-06964-5)
Supplement: Supplementary file 2 — Additional file 2: Fig. S2 Morphological changes and caspase activation in A549 cells treated with chemotherapeutics. (A) Analysis of cell phenotype assessed by flow cytometry. Population 1 indicates normal cell morphology. Reduced cell size (low FSC) with increased intracellular granularity (high SSC) is suggestive of early apoptosis (population 3); high intracellular granularity (high SSC) suggests cytoplasmic vacuolization, as observed after autophagy activation (population 2); and cell debrie suggests late apoptosis, which is characterized by cell fragmentation (population 4). A scheme showing these populations is shown on the right. (B) Cell area measured through images from immunocytochemistry. (C) Representative images of nuclear staining with DAPI (magnification: 200x). (D) Representative plots of active caspase 3 versus FSC. Red area delimits the population of cells with small size (i.e. cell shrinkage, a typical change of early apoptotic cells) and active caspase 3. Numbers represent the percentage of cells inside the red area ± SEM. Cisplatin 80 μM was used as a positive control (C+). [file 12885_2020_6964_MOESM2_ESM.pdf]

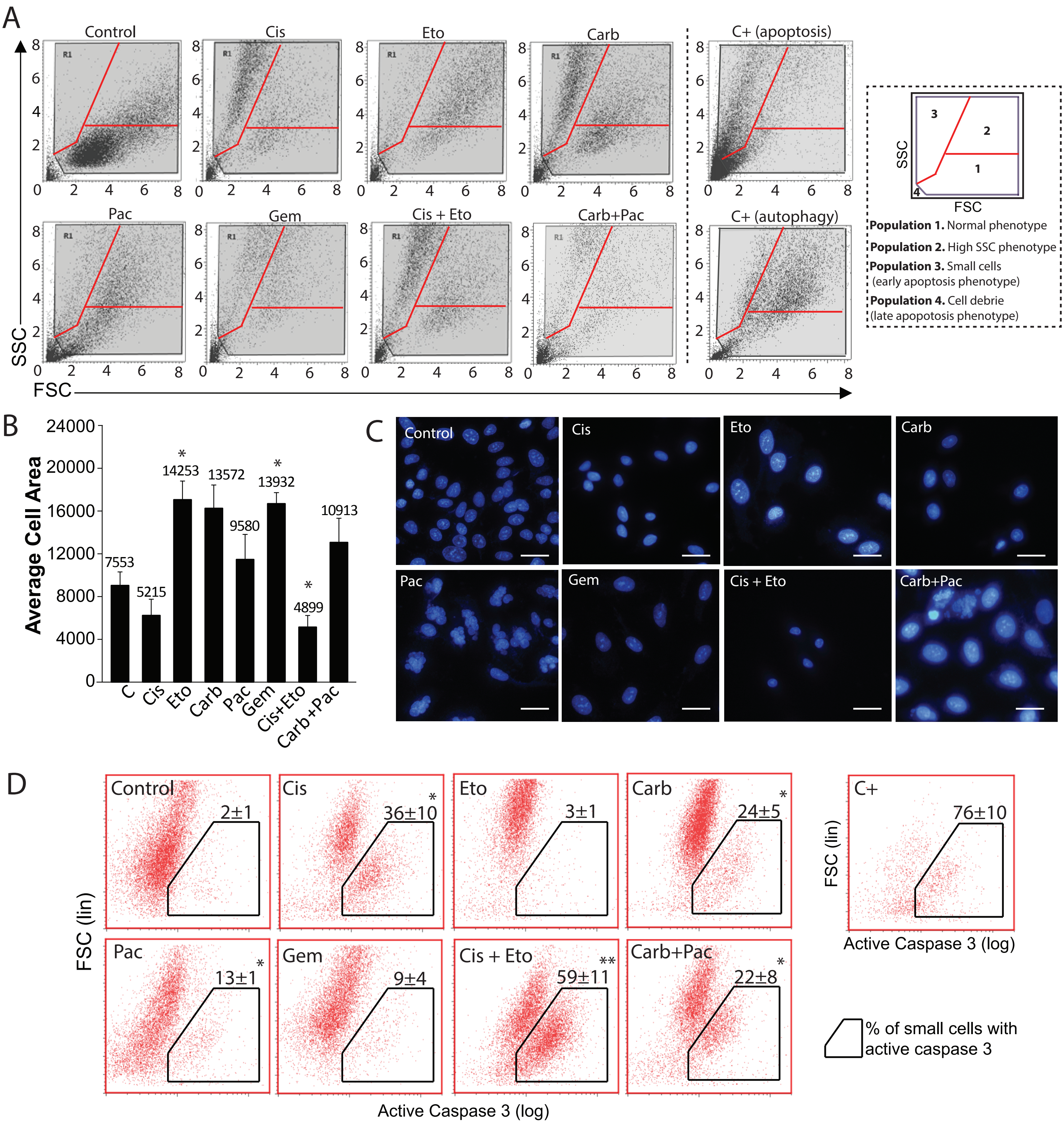

Figure S2. Morphological changes and caspase activation in A549 cells treated with chemotherapeutics. (A) Analysis of cell phenotype assessed by flow cytometry. Population 1 indicates normal cell morphology. Reduced cell size (low FSC) with increased intracellular granularity (high SSC) is suggestive of early apoptosis (population 3); high intracellular granularity (high SSC) suggests cytoplasmic vacuolization, as observed after autophagy activation (population 2); and cell debrie suggests late apoptosis, which is characterized by cell fragmentation (population 4). A scheme showing these populations is shown on the right. (B) Cell area measured through images from immunocytochemistry. (C) Representative images of nuclear staining with DAPI (magnification: 200x). (D) Representative plots of active caspase 3 versus FSC. Red area delimits the population of cells with small size (i.e. cell shrinkage, a typical change of early apoptotic cells) and active caspase 3. Numbers represent the percentage of cells inside the red area ± SEM. Cisplatin 80µM was used as a positive control (C+).
