# Supplementary material for: Damage-associated molecular patterns (DAMPs) related to immunogenic cell death are differentially triggered by clinically relevant chemotherapeutics in lung adenocarcinoma cells
Source: BMC Cancer. 2020 May 26;20:474. doi: 10.1186/s12885-020-06964-5 (PMC7251700; doi:10.1186/s12885-020-06964-5)
Supplement: Supplementary file 3 — Additional file 3: Fig. S3 Nuclear and LC3 co-staining. Representative images from each treatment are shown. Double arrowheads: nuclei classified as small and regular in the NMA; Arrows – nuclei classified as large in the NMA; Single arrowhead: nuclei classified as normal in NMA. [file 12885_2020_6964_MOESM3_ESM.pdf]

Figure S3

Solari & Filippi-Chiela et al

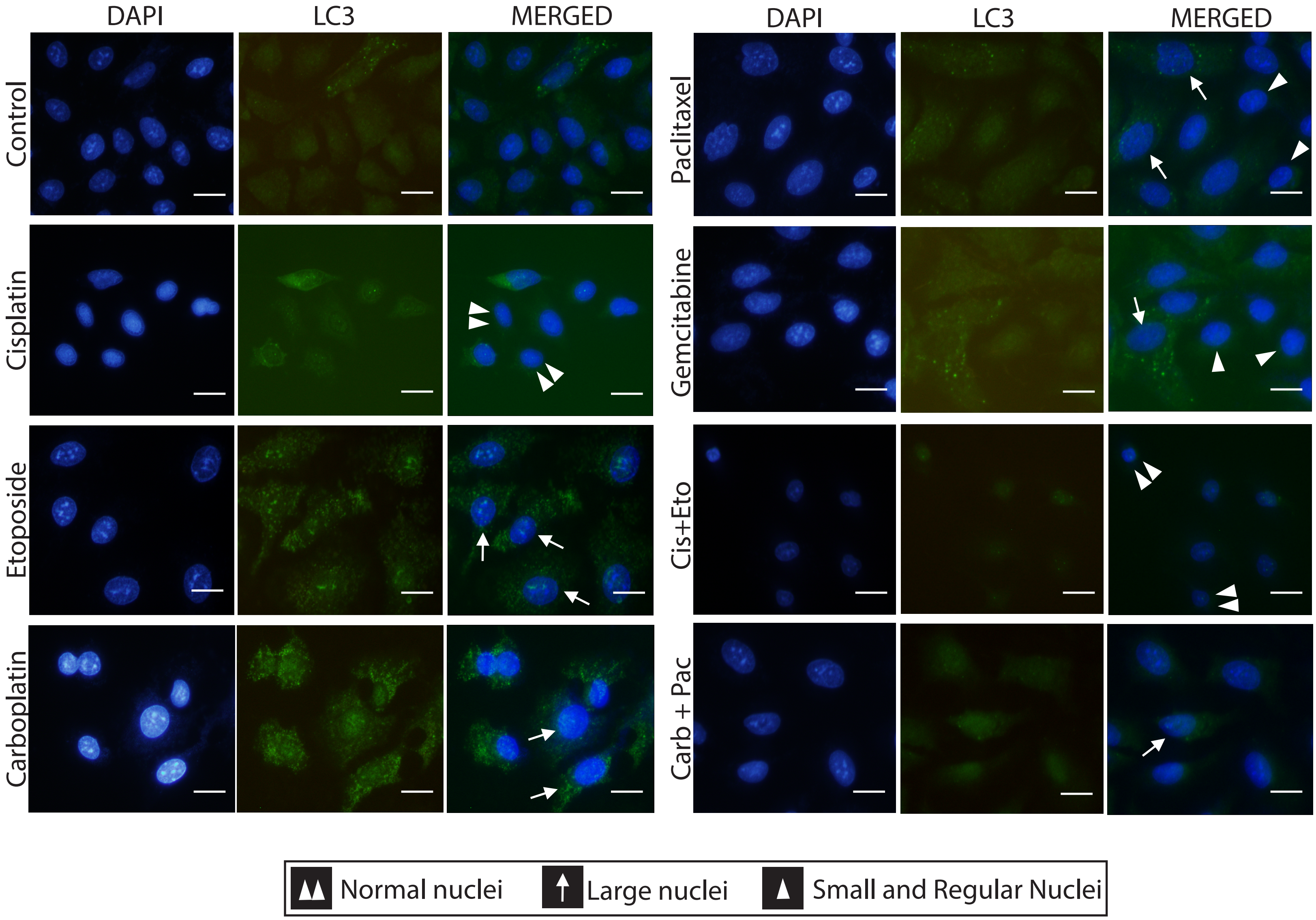

Figure S3. Nuclear and LC3 co-staining. Representative images from each treatment are shown. Double arrowheads: nuclei classified as small and regular in the NMA; Arrows – nuclei classified as large in the NMA; Single arrowhead: nuclei classified as normal in NMA.
