# Supplementary material for: Damage-associated molecular patterns (DAMPs) related to immunogenic cell death are differentially triggered by clinically relevant chemotherapeutics in lung adenocarcinoma cells
Source: BMC Cancer. 2020 May 26;20:474. doi: 10.1186/s12885-020-06964-5 (PMC7251700; doi:10.1186/s12885-020-06964-5)
Supplement: Supplementary file 4 — Additional file 4: Fig. S4 Calreticulin (CRT) exposure and its correlation with cell area. (A) Representative histograms of cell count and CRT exposure in the cell surface, as obtained by flow cytometry. *p < 0.05, **p < 0.01 and ***p < 0.001 in relation to control. (B) Dot plots for CRT levels and cell size (FSC). (C) Correlation between CRT margination and cell area, measured from calreticulin immunocytochemistry (Fig. 3B). *p < 0.05, **p < 0.01 in relation to control. [file 12885_2020_6964_MOESM4_ESM.pdf]

Figure S4 Solari & Filippi-Chiela et al

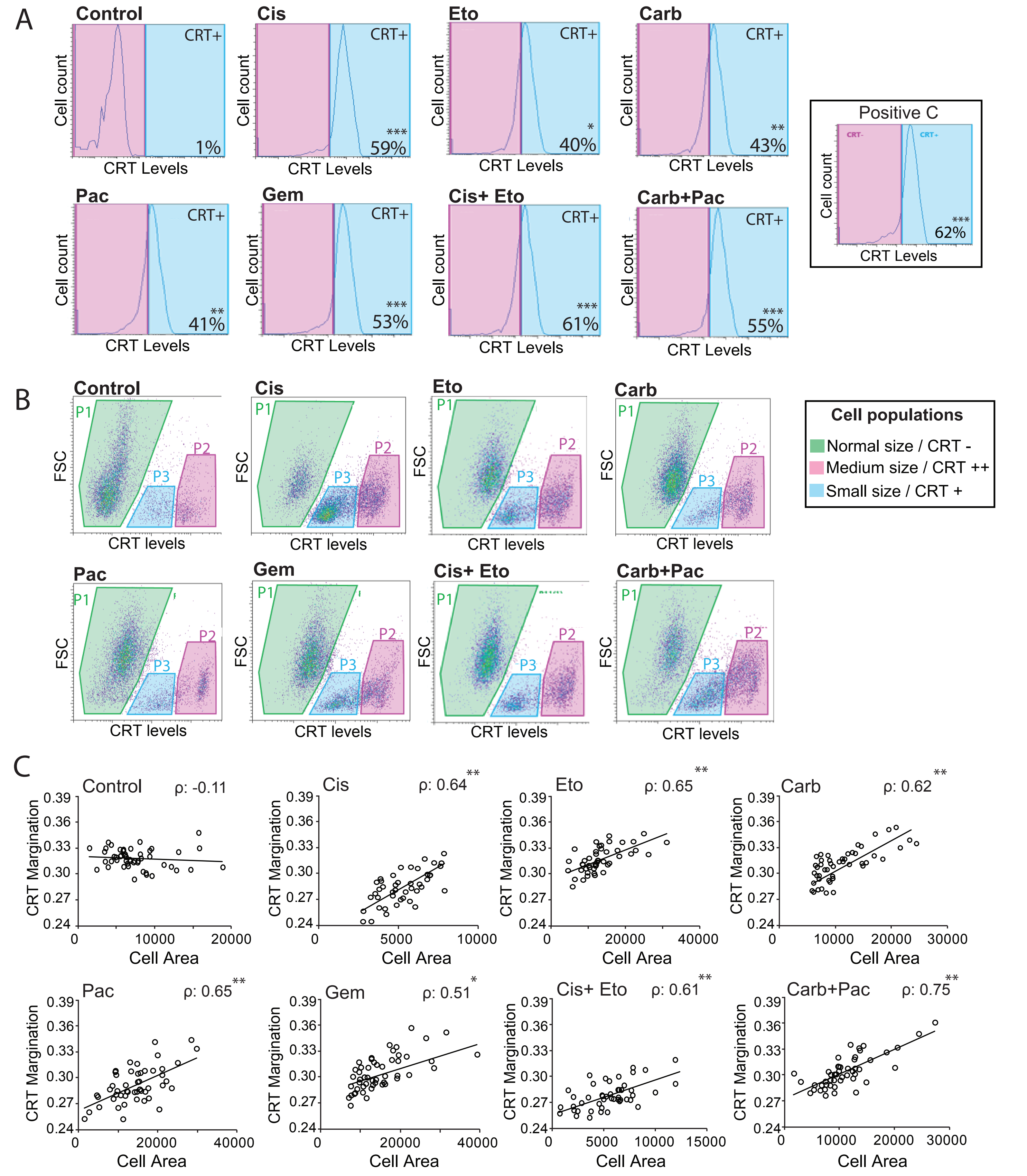

Figure S4 - Calreticulin (CRT) exposure and its correlation with cell area. (A) Representative histograms of cell count and CRT exposure in the cell surface, as obtained by flow cytometry. *p<0.05, **p<0.01 and ***p<0.001 in relation to control. (B) Dot plots for CRT levels and cell size (FSC). (C) Correlation between CRT margination and cell area, measured from calreticulin immunocytochemistry (Fig. 3B). *p<0.05, **p<0.01 in relation to control.
