# Supplementary material for: Damage-associated molecular patterns (DAMPs) related to immunogenic cell death are differentially triggered by clinically relevant chemotherapeutics in lung adenocarcinoma cells
Source: BMC Cancer. 2020 May 26;20:474. doi: 10.1186/s12885-020-06964-5 (PMC7251700; doi:10.1186/s12885-020-06964-5)
Supplement: Supplementary file 5 — Additional file 5: Fig. S5 Influence of CRT levels in the prognosis and staging of NSCLC. (A) Kaplan-Meier survival curve to patients expressing high or low levels of CRT in the TCGA cohort. (B) Dead/live ratio of patients for grade i-iv tumors according to CRT levels. (C to F) Influence of CRT levels on the outcome (i.e. dead or alive) of patients with stage i to iv NSCLC. [file 12885_2020_6964_MOESM5_ESM.pdf]

Figure S5 Solari & Filippi-Chiela et al

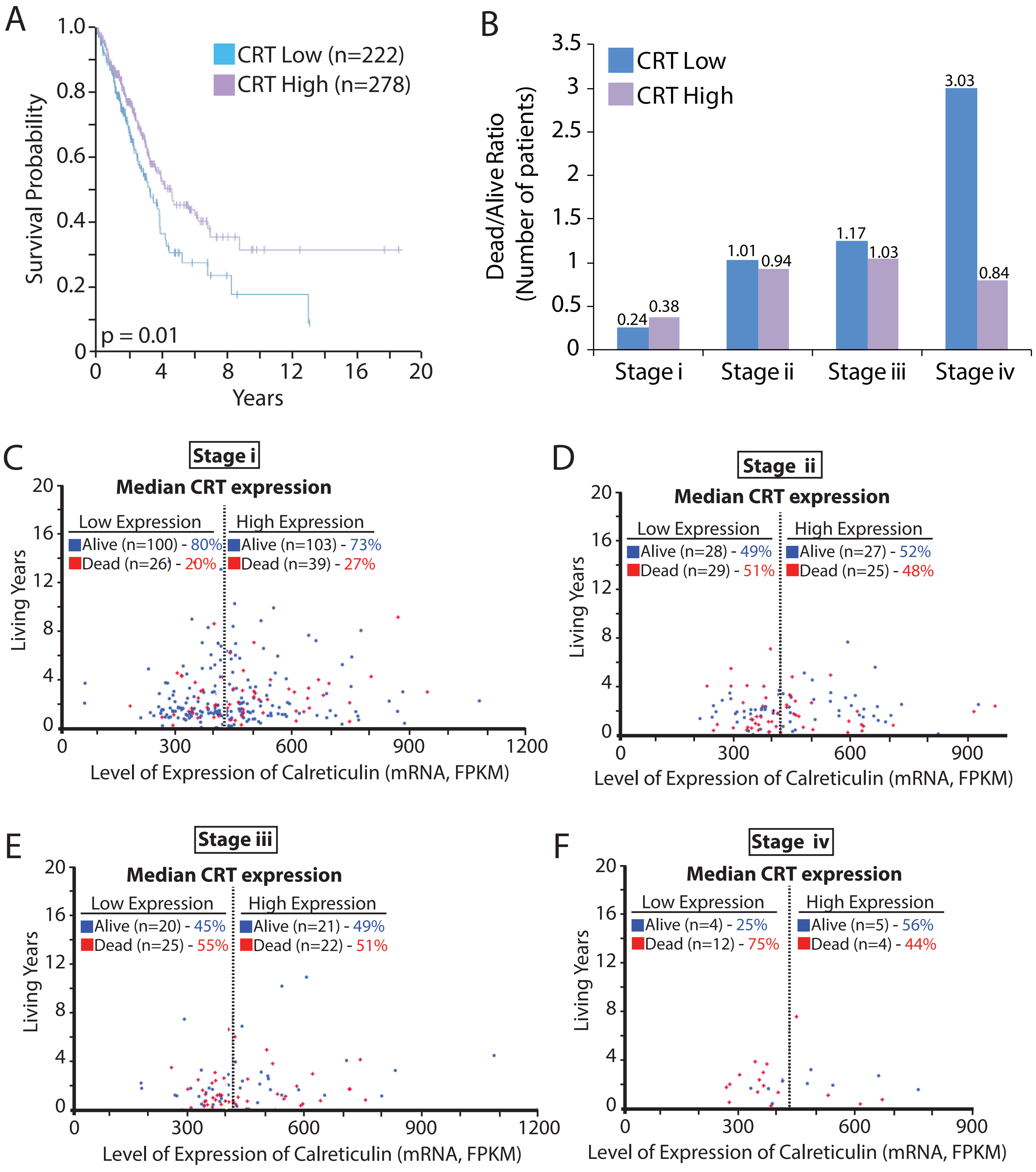

Figure S5 - Influence of CRT levels in the prognosis and staging of NSCLC. (A) Kaplan-Meier survival curve to patients expressing high or low levels of CRT in the TCGA cohort. (B) Dead/live ratio of patients for grade i-iv tumors according to CRT levels. (C to F) Influence of CRT levels on the outcome (i.e. dead or alive) of patients with stage i to iv NSCLC.
