# Supplementary material for: Damage-associated molecular patterns (DAMPs) related to immunogenic cell death are differentially triggered by clinically relevant chemotherapeutics in lung adenocarcinoma cells
Source: BMC Cancer. 2020 May 26;20:474. doi: 10.1186/s12885-020-06964-5 (PMC7251700; doi:10.1186/s12885-020-06964-5)
Supplement: Supplementary file 6 — Additional file 6: Fig. S6 Correlation matrix to all measurements assessed. ρ coef. Represent the pearson correlation coefficients. Red and green boxes correspond to all negative or positive significant correlations, respectively. Abbreviatures: ‘% Annex+/PI- cells’ - % of annexin+/Propidium Iodide negative cells; ‘% CRT+ cells’ - % of calreticulin-positive cells; ‘% AO+ cells’ - % of acridine orange-positive cells; ‘ATP’ - levels of extracellular ATP; ‘HMGB1’ - levels of extracellular HMGB1; ‘CRT Marg.’ - Margination of Calreticulin; ‘%LR, %N, %SR and %Irreg’ - percentage of ‘Large and Regular’, ‘Normal’, ‘Small and Regular’ and ‘Irregular’ nuclei from NMA analysis; ‘Nuc Area’ - nuclear area. [file 12885_2020_6964_MOESM6_ESM.pdf]

		Cell number	% Annex+/PI- cells	% AO+ cells	% CRT+ cells	ATP	HMGB1	CRT Marg.	% LC3+ cells	% Casp3+ cells	% LR	%N	%SR	%Irreg	Cell Area	Nuc Area
Cell number	ρ coef.	1	-,750*	-,731*	-,873**	-,772*	-,461	-,360	-,615	-770	-,299	,780*	-,500	-,152	-,083	-,045
	Sig.		,032	,039	,005	,025	,251	,382	,105	,049	,473	,022	,208	,720	,845	,915
% Annex+/PI- cells	ρ coef.		1	,216	,814*	,882**	,791*	,299	,455	,852**	-,175	-,293	,881**	-,142	-,135	-,483
	Sig.			,608	,014	,004	,020	,472	,257	,007	,679	,482	,004	,737	,749	,226
% AO+ cells	ρ coef.			1	,610	,315	-,172	,085	,531	,179	,778*	-,890**	-,188	,152	,516	,630
	Sig.				,108	,447	,684	,841	,176	,671	,023	,003	,655	,719	,191	,094
% CRT+ cells	ρ coef.				1	,921**	,628	,436	,342	,571	,277	-,757*	,503	,160	-,040	,071
	Sig.					,001	,096	,280	,408	,139	,507	,030	,204	,705	,925	,868
ATP	ρ coef.						,795*	,583	,215	,672	,059	-,538	,686	,023	-,374	-,168
	Sig.						,018	,129	,609	,068	,890	,169	,060	,957	,361	,690
HMGB1	ρ coef.						1	,543	,098	,517	-,517	-,101	,857**	,238	-,557	-,557
	Sig.							,164	,817	,190	,190	,812	,007	,571	,151	,152
CRT Marg.	ρ coef.							1	,099	,043	,087	-,338	,227	,139	-,734*	,158
	Sig.								,816	,920	,837	,413	,589	,742	,038	,709
% LC3+ cells	ρ coef.								1	,429	,104	-,250	,325	-,159	,384	-,084
	Sig.									,289	,807	,551	,433	,707	,348	,844
% Casp3+ cells	ρ coef.									1	-,141	-,197	,816*	-,300	-,046	-,552
	Sig.										,739	,641	,014	,470	,914	,156
% LR	ρ coef.										1	-,695	-,591	-,154	,365	,878**
	Sig.											,056	,123	,716	,374	,004
%N	ρ coef.											1	,076	-,377	-,175	-,610
	Sig.												,858	,358	,678	,109
%SR	ρ coef.												1	-,070	-,330	-,810*
	Sig.													,869	,425	,015
%Irreg	ρ coef.													1	-,063	,184
	Sig.														,883	,662
Cell Area	ρ coef.														1	,264
	Sig.															,527
Nuc Area	ρ coef.															
	Sig.															

Figure S6 - Correlation matrix to all measurements assessed. ρ coef. represents the pearson correlation coefficient. Red and green boxes correspond to all negative or positive significant correlations, respectively. Abbreviatures: ‘% Annex+/PI- cells’ - % of annexin+/Propidium Iodide negative cells; ‘% CRT+ cells’ - % of calreticulin-positive cells; ‘% AO+ cells’ - % of acridine orange-positive cells; ‘ATP’ - levels of extracellular ATP; ‘HMGB1’ - levels of extracellular HMGB1; ‘CRT Marg.’ - Margination of Calreticulin; ‘%LR, %N, %SR and %Irreg’ - percentage of ‘Large and Regular’, ‘Normal’, ‘Small and Regular’ and ‘Irregular’ nuclei from NMA analysis; ‘Nuc Area’ - nuclear area.
