# Supplementary material for: Damage-associated molecular patterns (DAMPs) related to immunogenic cell death are differentially triggered by clinically relevant chemotherapeutics in lung adenocarcinoma cells
Source: BMC Cancer. 2020 May 26;20:474. doi: 10.1186/s12885-020-06964-5 (PMC7251700; doi:10.1186/s12885-020-06964-5)
Supplement: Supplementary file 7 — Additional file 7: Fig. S7 Influence of HMGB1 levels in the prognosis of NSCLC. Kaplan–Meier survival analysis according to HMGB1 levels. [file 12885_2020_6964_MOESM7_ESM.pdf]

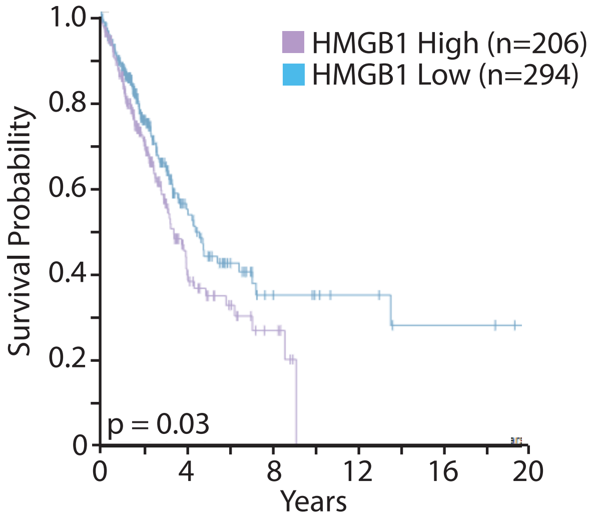

Figure S7 - Influence of HMGB1 levels in the prognosis of NSCLC. Kaplan–Meier survival analysis according to HMGB1 levels.
